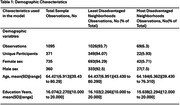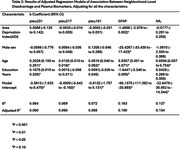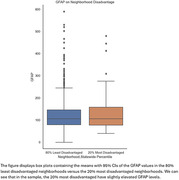# Neighborhood disadvantage and ADRD pathology assessed with plasma biomarkers

**DOI:** 10.1002/alz.092869

**Published:** 2025-01-09

**Authors:** Samriddhi Dube, W. Ryan Powell, William R. Buckingham, Kao Lee Yang, Sterling C. Johnson, Nicholas J. Ashton, Kaj Blennow, Henrik Zetterberg, Amy J.H. Kind, Barbara B. Bendlin

**Affiliations:** ^1^ Center for Health Disparities Research, University of Wisconsin School of Medicine and Public Health, Madison, WI USA; ^2^ Department of Medicine, Geriatrics Division, University of Wisconsin School of Medicine and Public Health, Madison, WI USA; ^3^ Center for Health Disparities Research, Department of Medicine, School of Medicine and Public Health (SMPH), University of Wisconsin‐Madison, Madison, WI USA; ^4^ Wisconsin Alzheimer's Disease Research Center, University of Wisconsin School of Medicine and Public Health, Madison, WI USA; ^5^ Wisconsin Alzheimer’s Disease Research Center, University of Wisconsin‐Madison School of Medicine and Public Health, Madison, WI USA; ^6^ Wisconsin Alzheimer's Institute, University of Wisconsin School of Medicine and Public Health, Madison, WI USA; ^7^ Geriatric Research Education and Clinical Center, William S. Middleton Memorial Veterans Hospital, Madison, WI USA; ^8^ Department of Psychiatry and Neurochemistry, Institute of Neuroscience and Physiology, The Sahlgrenska Academy, University of Gothenburg, Mölndal, Gothenburg Sweden; ^9^ Department of Psychiatry and Neurochemistry, Institute of Neuroscience and Physiology, The Sahlgrenska Academy, University of Gothenburg, Mölndal Sweden; ^10^ Department of Neurodegenerative Disease, UCL Queen Square Institute of Neurology, University College London, London, ‐ United Kingdom; ^11^ UK Dementia Research Institute, University College London, London United Kingdom; ^12^ Hong Kong Center for Neurodegenerative Diseases, Clear Water Bay Hong Kong; ^13^ Wisconsin Alzheimer's Disease Research Center, School of Medicine and Public Health, University of Wisconsin‐Madison, Madison, WI USA; ^14^ Wisconsin Alzheimer's Institute, University of Wisconsin‐Madison, Madison, WI USA

## Abstract

**Background:**

Neighborhood disadvantage as measured by the Area Deprivation Index (ADI) is associated with Alzheimer’s disease (AD) pathology at autopsy, as well as neuroimaging measures of neurodegeneration. Plasma biomarker assays have emerged as an accessible tool for evaluating ADRD pathology, opening up the possibility of better understanding the effect of neighborhood disadvantage on brain disease among a broader group of research participants. In this study, we evaluated whether neighborhood‐level socioeconomic disadvantage is associated with plasma‐based biomarkers of Alzheimer's disease and related pathology.

**Method:**

The participants were 371 individuals enrolled in the Wisconsin Registry for Alzheimer’s Prevention (WRAP) study and data included 1095 unique plasma measurements of the participants. EDTA plasma was assayed for pTau 217, pTau181 and pTau231, neurofilament light chain protein (NfL), and glial fibrillary acidic protein (GFAP). Participants’ addresses, as on the plasma measurement date, were geolinked to their statewide ranking of neighborhood disadvantage using a time‐concordant ADI. We compared participants living in the 20% most disadvantaged areas based on state residence (ADI of 9 or 10) to all other participants (80% less disadvantaged, ADI of 1‐8) by fitting adjusted linear regression models. We used the age, sex and years of education of the participants at the point of sample collection as characteristics.

**Result:**

Living in the 20% most disadvantaged neighborhood was associated with higher GFAP concentration, an effect that was marginally significant (b = ‐1.4898; 95% CI; ‐2.978 to ‐0.002; P = 0.050). No significant effects of ADI were observed on pTau 217, pTau181, pTau231, or NfL.

**Conclusion:**

Living in the most disadvantaged context was associated with elevated GFAP, a marker of astroglial activation. Elevated GFAP has previously been associated with amyloid pathology and neuroinflammation. The absence of significant associations with ptau biomarkers here may suggest non‐amyloid related processes, although this study may also be underpowered to detect relationships with amyloid, and further investigation is needed. There is an opportunity for future research to gain insight further into the mechanisms responsible for this association. Leveraging plasma biomarkers, future studies can provide a mechanistic understanding for the effects of contextual disadvantage on ADRD development.